# Ecotoxicity evaluation of thymol in tunneller dung beetles (Coleoptera: Geotrupidae) as a natural alternative to ecotoxic anthelmintics

**DOI:** 10.1007/s10646-026-03124-6

**Published:** 2026-07-02

**Authors:** Vieyle Cortez, Estela González-Rodríguez, José R. Verdú, Antonio J. Ortiz, Rocío Rosa-García

**Affiliations:** 1 Research Institute CIBIO (Centro Iberoamericano de la Biodiversidad), Universidad de Alicante, Science Park, Alicante, Spain; 2 Departamento de Enfermería, Universidad de Alicante, Alicante, Spain; 3 Departamento de Química Inorgánica y Química Orgánica, Universidad de Jaén, EPS de Linares, Jaén, Spain; 4 SERIDA−Servicio Regional de Investigación y Desarrollo Agroalimentario, Villaviciosa, Asturias Spain

**Keywords:** Geotrupidae, Ecotoxicology, Anthelmintics, Ivermectin, Phytochemical anthelmintics, Thymol

## Abstract

Synthetic anthelmintics widely used in livestock production are largely excreted unchanged in faeces, where they can exert sublethal and lethal effects on non-target organisms such as dung beetles. Phytochemical compounds, including thymol (THY), have been proposed as environmentally compatible alternatives; however, information on their ecotoxicological safety across different dung beetle taxa remains limited. We assessed the sublethal effects of dietary thymol on *Thorectes lusitanicus* (Coleoptera: Geotrupidae), a flightless tunneller dung beetle considered functionally sensitive to veterinary drug residues. Antennal sensory responses were evaluated using electroantennography, and immune function was assessed by measuring haemolymph total protein content, phenoloxidase and prophenoloxidase activities. Ivermectin was included as a reference compound due to its well-documented ecotoxic effects on dung beetles. Thymol ingestion did not affect antennal olfactory responses or immune parameters across a wide range of concentrations, and no neurotoxic symptoms were observed. Significant inhibitory effects were detected only at concentrations substantially higher than thymol residues measured in cattle dung following dietary supplementation. In contrast, ivermectin significantly impaired olfactory function and induced clear neurotoxic symptoms. Overall, these results indicate that thymol is unlikely to cause ecotoxicologically relevant sublethal effects in tunneller dung beetles under the exposure scenarios evaluated. Our findings suggest that thymol-based phytochemical anthelmintics may represent environmentally compatible alternatives to conventional veterinary drugs.

## Introduction

Veterinary medicinal products (VMPs) are used worldwide to control endo- and ectoparasites in livestock. Due to their documented environmental impacts, several major classes of VMPs are recognised as posing significant ecological risks. Among these, macrocyclic lactones (MLs) represent an important group of synthetic anthelmintics extensively used in both grazing and intensively managed livestock since the 1980s (Campbell 1989). These compounds are largely excreted unmetabolised in faeces, accounting for approximately 90% of the administered dose, at concentrations that are toxic to non-target invertebrates, including functionally important coprophagous insects such as dung beetles (Wall and Strong 1987; Floate et al. 2005; Canga et al. 2009; de Souza and Guimarães 2022). This toxicity arises because these compounds act by disrupting signal transmission in the nerve and muscle cells of invertebrates, including nematodes and arthropods, ultimately leading to paralysis and death (Wolstenholme and Rogers 2005).

MLs raise substantial environmental concerns by contributing to ecosystem contamination and biodiversity loss (Lumaret and Errouissi 2002; Beynon et al. 2012; Lumaret et al. 2012). The use of these compounds can adversely affect rangeland health, as dung beetles provide essential ecosystem services, including dung decomposition and the recycling of nutrients from dung into the soil (Floate et al. 2005; Nichols et al. 2008; Slade et al. 2016; DeCastro-Arrazola et al. 2022). Numerous studies have demonstrated strong negative impacts of MLs, particularly ivermectin (IVM), on dung beetle physiology, reproduction and survival. Depending on their concentration, faecal residues may induce sublethal, pre-lethal or lethal effects, impairing key physiological and behavioural processes in dung beetles (Wall and Strong 1987; Roncalli 1989; Sommer and Nielsen 1992; Krüger and Scholtz 1997; Dadour et al. 2000; Wardhaugh et al. 2001; Steel and Wardhaugh 2002; Iwasa et al. 2007; Lumaret et al. 2012; Wall and Beynon 2012; Jacobs and Scholtz 2015; Adler et al. 2016; González-Tokman et al. 2017; Martínez et al. 2017; Verdú et al. 2015, 2018a, b, 2020a ,b; Villada-Bedoya et al. 2019; Finch et al. 2020; Junco et al. 2021; Mackenzie et al. 2022; Su et al. 2023; Urrutia et al. 2025). Recent evidence has further shown that IVM can be biomagnified in adult dung beetles (Coleoptera: Geotrupidae) exposed to non-lethal doses through dietary ingestion (Verdú et al. 2020a).

There is a growing consensus that the widespread use of MLs should be reduced in extensive livestock systems. Anthelmintics administration not only affects livestock health but also compromises the integrity of ecological processes that underpin livestock production itself, as dung beetles are key providers of ecosystem services. In particular, they play a crucial role in nutrient cycling, soil aeration, and parasite suppression in livestock systems (Losey and Vaughan 2006; Nichols et al. 2008; Beynon et al. 2015). Therefore, there is an urgent need to develop sustainable and environmentally compatible alternatives for controlling parasitic infections that minimize negative effects on dung beetles.

Medicinal plants and commercial phytotherapeutic products, including formulations based on plant extracts, homeopathy, minerals and trace elements, are increasingly studied and considered viable alternative parasite-control strategies as resistance to conventional anthelmintics has increased (García Romero 2008; Mayer et al. 2014; French 2018; Hikal et al. 2021). Thymol (2-isopropyl-5-methylphenol; THY), the major active constituent of essential oils from several *Thymus* and *Origanum* species (Escobar et al. 2020; Hikal et al. 2021), has well-known anthelmintic properties and is widely used in phytotherapeutic treatments against parasitic infections (Ferreira et al. 2016; Salehi et al. 2018; Kheirandish et al. 2020; Kowalczyk et al. 2020; Biswal and Pazhamalai 2021; Shehata et al. 2022; Dardona et al. 2024). Several studies indicate that THY can interfere with parasite physiology, including gastrointestinal nematodes and protozoa, through mechanisms comparable to those of MLs, inducing structural and functional alterations in parasite cells (Hibbs and Gouaux 2011; Lynagh et al. 2014; Ferreira et al. 2016; Meeran et al. 2017; Domínguez-Uscanga et al. 2021; Pérez Gaudio et al. 2025). In addition, THY has been shown to reduce pathogen loads, odour emissions and greenhouse gas production from livestock dung (Varel 2002; Varel and Wells 2007; Yu et al. 2020; Benchaar et al. 2021), further supporting its potential role within environmentally sustainable parasite control strategies.

THY has emerged as a promising alternative to MLs, mainly due to its low residual concentrations in cattle faeces after administration of commercial formulations (Meeran et al. 2017; Kowalczyk et al. 2020; Hikal et al. 2021; Verdú et al. 2023) and its demonstrated safety for dung beetles in standardised ecotoxicological tests (Verdú et al. 2023). Recent studies have shown that phytochemical anthelmintics, including THY, do not pose ecotoxic risks to the dung beetle *Ateuchetus cicatricosus* (Coleoptera: Scarabaeidae) even at extremely high concentrations nearly 1000 times higher than those expected under field conditions (Verdú et al. 2023). Nevertheless, it remains premature to assume negligible risks for all dung beetle taxa. Several studies indicate that the sensitivity of dung beetles to MLs residues varies markedly among species and functional groups (Beynon et al. 2012; Jacobs and Scholtz 2015; Manning et al. 2018; Martínez et al. 2018; Ishikawa and Iwasa 2020). Field studies from Brazil and the Colombian Caribbean further suggest that tunneller dung beetles are often the most vulnerable functional groups in livestock systems treated with IVM, compared with rollers (Sands and Wall 2018; Correa et al. 2022; Tovar et al. 2023). However, the mechanistic basis of these differences in sensitivity of dung beetles to ML residues remains poorly understood, and even less is known about their response to more sustainable alternatives.

In this study, we evaluated the effects of THY on the tunneller beetle *Thorectes lusitanicus* (Coleoptera: Geotrupidae) using standardised ecotoxicity tests, including electroantennography (EAG) and immune system assays (prophenoloxidase, phenoloxidase, and total protein content). *T. lusitanicus* is a flightless, telephagic tunneller endemic to the southern Iberian Peninsula (Martín-Piera and López-Colón 2000), widely used as a model organism in physiological studies due to its ease of maintenance and the relatively large volume of haemolymph it produces (Verdú et al. 2010, 2013, 2020a).

The aim of this study was to assess the sublethal effects of THY on *T. lusitanicus* using two physiological endpoints. First, given that the olfactory system plays a central role in dung beetle ecology (Urrutia et al. 2022), we quantified antennal sensory responses using electroantennography. This neurophysiological technique measures the electrical response of insect antennae to olfactory stimuli. It provides a sensitive indicator of olfactory function and allows the detection of sublethal effects of chemical exposure, making it a suitable tool to assess biological impacts (Verdú et al. 2015, 2018b). Second, given the key role of phenoloxidase (PO) in insect innate immunity and melanisation (Schmid-Hempel 2005; González-Santoyo and Córdoba-Aguilar 2012), we evaluated changes in immune function following THY exposure.

## Materials and methods

### Test organisms

This study used the dung beetle *Thorectes lusitanicus* as the model species due to its high local abundance (> 30 individuals frequently collected per dung pat), relatively large body mass (~ 1.5 g fresh weight per individual), and suitability for laboratory rearing. In addition, its high haemolymph volume has facilitated its use in previous ecophysiological studies (Verdú et al. 2013, 2020a). Specimens were collected in La Sauceda, Los Alcornocales Natural Park, southern Spain (36°31′54″N, 5°34′29″W), an area free of ivermectin contamination. Live individuals were captured using cow dung-baited pitfall traps and subsequently transported to the laboratory. During transport to the laboratory, individuals were kept in plastic containers (60 × 40 × 40 cm) containing moss and dried Quercus leaves as substrate to minimize handling stress. Upon arrival, beetles were transferred to terraria lined with moist paper and maintained in a climate-controlled chamber at 18 ± 1 °C, 65% relative humidity, and a 14:10 h light: dark photoperiod. Individuals were maintained under these conditions and fed ivermectin-free cow dung for one week. Thereafter, beetles were kept under identical environmental conditions for three additional days without food prior to experimentation.

Adult individuals of *T. lusitanicus* were identified based on external morphological characteristics using standard taxonomic keys for Geotrupidae (Baraud 1992). To standardise physiological condition, only mature individuals were selected based on external age-grading criteria (e.g., fore tibial abrasion and pronotal and elytral cuticle hardness), allowing selection of beetles of comparable age. Prior to treatment allocation, all individuals were sexed to ensure a 1:1 sex ratio in each experiment, individually marked, and weighed to obtain fresh body mass. All procedures complied with Spanish legislation concerning animal conservation and welfare.

### Fecal residue analysis

Prior to the ecotoxicity bioassays, thymol (THY) concentrations in cattle dung were quantified to determine the actual residue levels to which dung beetles would be exposed under experimental conditions. Dung samples used for residue analysis were collected from six cows belonging to the Asturian Mountain and Asturian Valley breeds. The experiment was carried out at the Carbayal Research Station (6° 53′ W, 43° 21′ N; Sierra de San Isidro, Illano, Asturias, Spain). Before the start of the trial, all cows were weighed and housed in separate enclosures. Three cows received, for seven consecutive days, a basal ration of herbage silage (5.6 kg head–1 day–1) supplemented with NEXT ENHANCE^®^ 150 (300 mg head^–1^ day^–1^), while another three cows were offered only the basal herbage silage diet (4.5 kg head^–1^ day^–1^) and served as the control group. The additive NEXT ENHANCE^®^ 150 (Novus International Inc., USA) contains THY (25% w/w) and carvacrol (25% w/w) as active ingredients. The ration was adjusted individually to 0.5% dry matter per kg live weight, and water was provided *ad libitum*. Daily feed intake was monitored for each animal. Dung samples (0.5 kg per cow) were collected after 1, 3 and 7 days of exposure to the supplemented diet, labelled, and stored at 1 °C until analysis.

Thymol (≥ 99% purity; Acros Organics, Lancaster, UK) was used for the preparation of both the stock solution and calibration standards. The stock solution (100 mg l⁻¹) was prepared dissolving THY in hexane, and calibration standards ranging from 0.01 to 20 mg l⁻¹ were obtained by serial dilution. To enhance partitioning of the lipophilic THY into the aqueous phase, aliquots of the stock solution were added to distilled water containing NaCl. Standards were prepared by placing 3 ml of water containing 0.6 g NaCl (20% w/v) into 10 ml glass vials sealed with magnetic caps and PTFE–silicone septa. A precise aliquot of the hexane stock solution was then added, and vials were equilibrated in a water bath at 25 °C to ensure stable partitioning prior to use. Volatile THY residues were sampled using solid-phase microextraction (SPME). The fibre was inserted through the septum and exposed to the vial headspace at 45 °C for 60 min to trap the hydroxylated monoterpene. After extraction, the fibre was desorbed in the gas chromatograph (GC) split/splitless injector at 250 °C for 5 min.

Bovine dung volatile collections were carried out with a field-portable SPME device fitted with a divinylbenzene–carboxen–polydimethylsiloxane fibre (DVB/CAR/PDMS; 50/30 µm grey notched; Supelco, USA). The fibre was conditioned according to the manufacturer’s instructions and cleaned after each injection by heating in the GC inlet at 270 °C for 60 min. Prior to volatile collection, 100 ml round-bottom glass flasks (Pobel S.L., Spain) were equilibrated for 1 h at 25 °C. Subsequently, 60 g of dung were transferred into a flask, which was immediately sealed with a rubber red 14/20 mm septum cap and placed in a water bath at 45 °C. After a 30 min solid–gas equilibration period to allow volatile compounds to reach the headspace, the septum was pierced with a needle and the SPME fibre was introduced and exposed to the headspace for 60 min. At the end of the extraction period, the fibre was retracted, removed from the flask, and inserted into the GC injection port, where desorption was performed at 250 °C for 5 min.

Analyses of dung volatiles were carried out on a gas chromatograph (Focus GC, Thermo, USA) coupled to a single-quadrupole mass spectrometer (Thermo DSQ II, USA). Electron impact ionisation was set at 70 eV, with an m/z range of 41–300 and a scan rate of 6 scans s⁻¹. The GC was equipped with a split/splitless injector fitted with an SPME glass liner (1 mm ID × 120 mm; Thermo Fisher, USA) and a DB-5 capillary column (30 m × 0.25 mm × 0.25 μm; JandW Scientific, Folsom, CA, USA) with helium as carrier gas at 1.2 ml min⁻¹. The injector temperature was 250 °C; the SPME fibre was held in splitless mode for 5 min before the split was opened, and then left in the injector for an additional 3 min. The oven temperature programme was: 60 °C (1 min), ramped at 5 °C min⁻¹ to 250 °C, and held at 250 °C for 15 min. The transfer line was maintained at 280 °C. Data acquisition and processing were performed using Xcalibur software (Thermo Fisher). Target compounds were identified by comparison of mass spectra with the Wiley 275 L library and by matching retention times and spectra with those of authentic standards.

### Experimental setup

Fresh cow dung free of veterinary medicinal product residues was obtained from untreated control cattle in the field experiment conducted at the Carbayal Research Station (see above). Approximately 20 kg of dung were homogenised with an electric mixer and stored under refrigeration at 1 °C until use.

For the ecotoxicity assay, six nominal concentrations of THY were selected: 0.1, 3, 10, 100, 300 and 1000 mg kg⁻¹ (fresh dung), covering a wide range of doses within a single experiment. THY (purity ≥ 98.5%; Sigma-Aldrich Co., St. Louis, USA) was dissolved in absolute ethanol (Sigma-Aldrich Co.), and 2 ml of each solution were added to 2 kg portions of fresh dung. Each treated dung batch was then mixed for 2 h using a kitchen mixer to ensure homogeneous distribution. Additionally, following previous findings (Verdú et al. 2023) and in accordance with OECD guidelines (Organisation for Economic Co-operation and Development, 2010), a concentration of 100 µg kg⁻¹ (fresh dung) of ivermectin (1% IVM; Ivomec^®^ Merial) in dung was used as a positive reference treatment, while untreated dung served as the negative control. The ivermectin treatment was prepared following the same preparation procedure. The exposure concentrations used in the assays were nominal concentrations prepared in fresh cow dung for both compounds. Based on previously validated protocols (Verdú et al. 2015, 2018b), no analytical verification of the concentrations of the compounds was performed. For the solvent control, absolute ethanol was added to the dung at the same volume as in the THY treatments. Residual ethanol was allowed to evaporate for 2 h before transferring the dung to the experimental units. All dung treatments were stored in sealed plastic containers at 1 °C to prevent desiccation until use.

During the experimental period, dung beetles were maintained individually in plastic containers (15 × 10 × 7 cm) lined with moist sterile paper as substrate. Containers were kept in a climate-controlled chamber at 18 °C (representative of optimal field temperatures), 85% relative humidity, and a 14:10 h light: dark photoperiod. Following established protocols (Verdú et al. 2015, 2023), dung was provided in 2 ml portions on 6 cm Petri dishes, ensuring that the dung did not come into contact with the substrate. This set-up allowed precise quantification of dung consumption and, consequently, THY intake per individual. Every three days, any uneaten dung was removed and its volume (ml) recorded, after which a fresh 2 ml portion from the corresponding treatment was supplied. Beetles were maintained on their respective dietary treatments for an average of 30 ± 5 days prior to the physiological bioassays.

To monitor potential symptoms associated with ingestion of the treatments (THY or IVM), two behavioural responses were assessed: (i) antennal reflex avoidance movements at the scape–pedicel joint, and (ii) coordinated walking ability. Individuals exhibiting normal antennal reflexes and locomotion were classified as healthy, whereas partial antennal paralysis and impaired coordination (ataxia) were recorded as indicative of neurotoxic effects, and the date of symptom onset was noted. Each treatment, including the IVM reference treatment and control groups, consisted of 20 independent replicates, with one individual of *T. lusitanicus* per container, which was considered the experimental unit. On the day of the physiological bioassays, beetles were weighed to obtain body mass, and the presence or absence of treatment-related symptoms was recorded.

### Electroantennography assays (EAG)

Olfactory responses were recorded from the antennae of *T. lusitanicus* using a Syntech EAG system (Syntech, Kirchzarten, Germany), following a methodology adapted from previously published protocols by Verdú et al. (2015, 2018b, 2023). The antenna was carefully excised at its base using fine dissection scissors. The base and terminal flagellomere of the antenna were connected to a pair of metal electrodes using a conductive adhesive gel (Spectra 360, Parker Laboratories, Fairfield, NJ, USA), and the prepared electrodes were inserted into the EAG probe (Type PRG-2). The antennal preparation was placed under a constant stream of humidified air (flow of 200 ml min^–1^) by an air stimulus controller (Syntech, CS-55). Total amplification was 10 × and a signal acquisition interface board (Syntech, IDAC-02) processed and digitized the amplified signals.

To test the EAG response, odour presentation followed procedures similar to those described in previous studies (Verdú et al. 2018b, 2023). A 1 µL aliquot of 10% aqueous ammonia (NH₃ 25%, CAS No. 1336-21-6; Sigma–Aldrich Co., diluted in H₂O), used as the test compound, was applied to a strip of filter paper (Whatman No. 1), which was then inserted into a glass Pasteur pipette (Fisher Scientific, Pittsburgh, PA, USA). Stimulation tests were conducted by delivering 2-s puffs of humidified air (200 ml min⁻¹) through the Pasteur pipette. The tip of the pipette was fitted into a side port of an L-shaped glass tube (130 mm in length × 12 mm in diameter). In each experiment, the antenna was first stimulated with the standard reference compound hexane (HPLC grade, Sigma–Aldrich Co.), followed by stimulation with the test odourant. At least 1 min was allowed between each puff for the recovery of antennal receptors. The resulting EAG amplitude was computed as the difference between the baseline level and the maximum reached during stimulation. Each recording was carried out independently in 6 replicates, with alternate intervals of 60 s to allow for antennal receptor recovery. Replicates were performed with different individuals (*n* = 12, for each treatment). The DC potential was recorded (Universal AC/DC probe), processed and analysed using GC-EAD 2015 software (version 1.2.5; Syntech).

### Immune assays

After the excision of the antenna for the EAG test, haemolymph was extracted by puncturing the cuticle on the dorsal side of the pronotum and gently squeezing the beetle, as previously described by Verdú et al. (2013). Haemolymph samples were placed into 0.5 ml Eppendorf tubes, protected from light, and frozen at − 80 °C in an ultrafreezer (SANYO Electric Co. Ltd, Japan) until analysis. Bioassays for phenoloxidase (PO) activity, prophenoloxidase (proPO) activity and the total protein content were conducted in accordance with the protocols described by Verdú et al. (2013), using a microplate method with slight modifications.

PO and proPO activities in the haemolymph were assayed spectrophotometrically by recording the oxidation of L-3,4-dihydroxyphenylalanine (L-DOPA; Sigma-Aldrich Co.). Briefly, 5 µl of haemolymph were diluted to 100 µl with phosphate-buffered saline (PBS) and subjected to two freeze–thaw cycles to disrupt cells and release their contents. The thawed haemolymph was then centrifuged at 20,000 × g for 5 min at 5 °C and used immediately. To measure PO activity, 50 µL of haemolymph solution were added to each well, followed by 50 µL of L-DOPA (20 mM). Each sample was tested in triplicate in a 96-well microplate (Nunc, Fisher France). Absorbance was measured at 492 nm at 30 s intervals for 40 min at 25 °C using a SPECTROstar^®^ Nano microplate reader (BMG Labtech GmbH). The linear phase was typically reached between 3 and 25 min. PO activity was expressed as the slope of the absorbance increase over a 10 min interval within the linear phase of the reaction. One unit of PO activity was defined as the amount of enzyme required to increase absorbance by 0.001 units per minute per ml of haemolymph. Data were processed using MARS software (BMG Labtech).

Phenoloxidase plays a key role in the immune defence of invertebrates. The active PO enzyme catalyses the production of several toxic products. To prevent immunopathological effects, PO is mainly stored as an inactive proenzyme, prophenoloxidase (proPO). proPO can be activated to PO upon infection or wounding, as well as by exposure to certain chemicals, heat, or mechanical agitation (Schmid-Hempel 2005; González-Santoyo and Córdoba‐Aguilar 2012; Verdú et al. 2013). To assess total PO activity, 200 µl of haemolymph solution were incubated with 5 µl of trypsine type IX-S (1.67 mg ml^–1^ in PBS; Sigma-Aldrich, Co) for 5 min at 25 °C. Subsequently, 200 µl of 20 mM L-DOPA were added, and total PO activity was measured as described above.

Protein concentration was estimated spectrophotometrically in triplicate using the Bradford method (1976) with Bradford reagent (Sigma-Aldrich, Co.). Briefly, haemolymph samples were centrifuged at 20,000 ×g for 5 min at 5 °C to separate the particulate material. The resulting supernatants were collected and diluted 1:100 with PBS (50 mM phosphate buffer, pH 7.4). Samples were prepared by adding 5 µL of haemolymph or standard to 250 µL of Bradford reagent in a 96 well microplate (Nunc, Fisher France) and incubated for 10 min at 25 °C. The protein-dye complex remained stable for approximately 60 min. Bovine serum albumin (BSA; Sigma-Aldrich, St. Louis, MO, USA.) was used to generate a standard curve comprising eight concentrations ranging from 0.1 to 1.4 mg ml^− 1^ of protein in buffer PBS. Absorbance was read at 595 nm using a microplate reader (SPECTROStar^®^ Nano, BMG Labtech, GmbH). Total protein concentrations were calculated by regression analysis of the standard curve using MARS software (BMG Labtech) and expressed as mg ml^− 1^.

### Statistical analysis

To evaluate the potential negative effects of different THY concentrations on antennal response and immune parameters (total protein content, PO and proPO activities), one-way ANOVAs were performed. Data normality was assessed using Kolmogorov–Smirnov tests (*P* > 0.05 for all variables). Although beetles were selected to minimise variability in body mass, all ecophysiological measurements were normalised by body weight (g). When significant effects were detected, Dunnett’s post hoc tests were applied to compare each treatment group with the control.

The physiological responses evaluated (antennal response, total protein content, proPO and PO activities) were selected as sensitive and ecologically relevant endpoints (Verdú et al. 2013, 2015, 2018b, 2023). THY concentrations capable of inhibiting or stimulating the selected ecophysiological variables were estimated from dose–response relationships using non-linear regression models (Verdú et al. 2023). Inhibition and stimulation thresholds were interpolated from dose–response curves and expressed as IC_50_ (the half maximal inhibitory concentration, or the concentration causing a 50% reduction or inhibition in the response variable) and EC_50_ (the half maximal effective concentration, or the concentration causing a 50% increase or stimulation in the response variable), respectively. All statistical analyses were performed using GraphPad Prism version 10 (San Diego, CA, USA).

## Results

### Fecal residues of thymol

Cows treated with NEXT ENHANCE^®^ 150 showed no feed refusal and consumed the full daily ration. The mean thymol (THY) concentration in faeces was 0.260 ± 0.071 mg kg^–1^, with no significant differences among sampling times (ANOVA: F = 0.090; d.f. = 2; *P* = 0.910).

### Ecotoxicity assays (EAG)

**Fig. 1:**
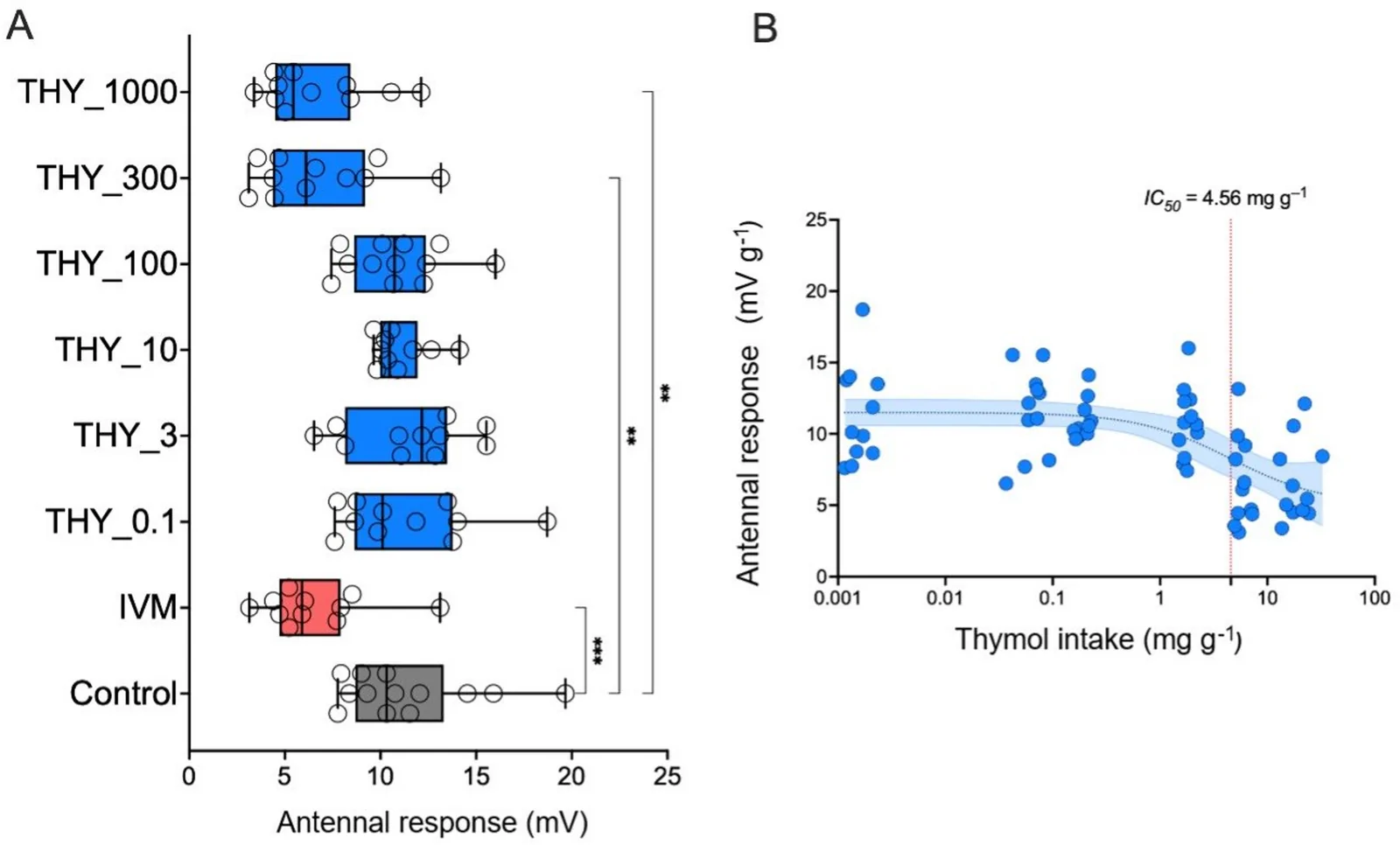
Effect of thymol intake on the olfactory system of *Thorectes lusitanicus*. (**A**) Antennal response (mV) to thymol (THY) at concentrations of 0.1, 3, 10, 100, 300, and 1000 mg kg⁻¹, compared to ivermectin (IVM; 100 µg kg⁻¹, positive control) and untreated dung (control; negative control). Colours represent the different compounds. Bars represent mean values ± standard deviation. Asterisks indicate significant differences compared to the control group (**P < 0.01; ***P < 0.001; Dunnett’s post hoc test). (**B**) Concentration–response curve for the antennal response by THY intake. The shaded area represents the 95% confidence interval. The IC_50_ value indicates the amount of THY ingested per gram of individual required to reduce the antennal response by 50% relative to control values

All control individuals remained alive and exhibited normal behaviour throughout the exposure period. THY ingestion at concentrations between 0.1 and 100 mg kg⁻¹ did not negatively affect antennal responses (Fig. 1A; ANOVA: F_7,82_ = 7.55, *P* < 0.001; Dunnett’s test: *P* > 0.99). However, significantly reduced responses were detected at higher concentrations (300 and 1000 mg kg^–1^; ANOVA: F_7,82_ = 7.55; Dunnett’s test: *P* = 0.0011). As expected, ivermectin (IVM; 100 µg kg⁻¹ fresh dung) produced a significant inhibitory effect on antennal sensitivity, confirming its role as a positive control (Fig. 1A; ANOVA: F_7,82_ = 7.55; *P* < 0.001; Dunnett’s test: *P* = 0.0008).

Dose–response modelling of antennal inhibition as a function of normalised THY intake yielded an IC_50_ value of 4.56 g g^–1^, indicating that relatively high exposure thresholds would be required to induce a 50% reduction in olfactory function (Fig. 1B).

### Ecotoxicity on the immune system

**Fig. 2:**
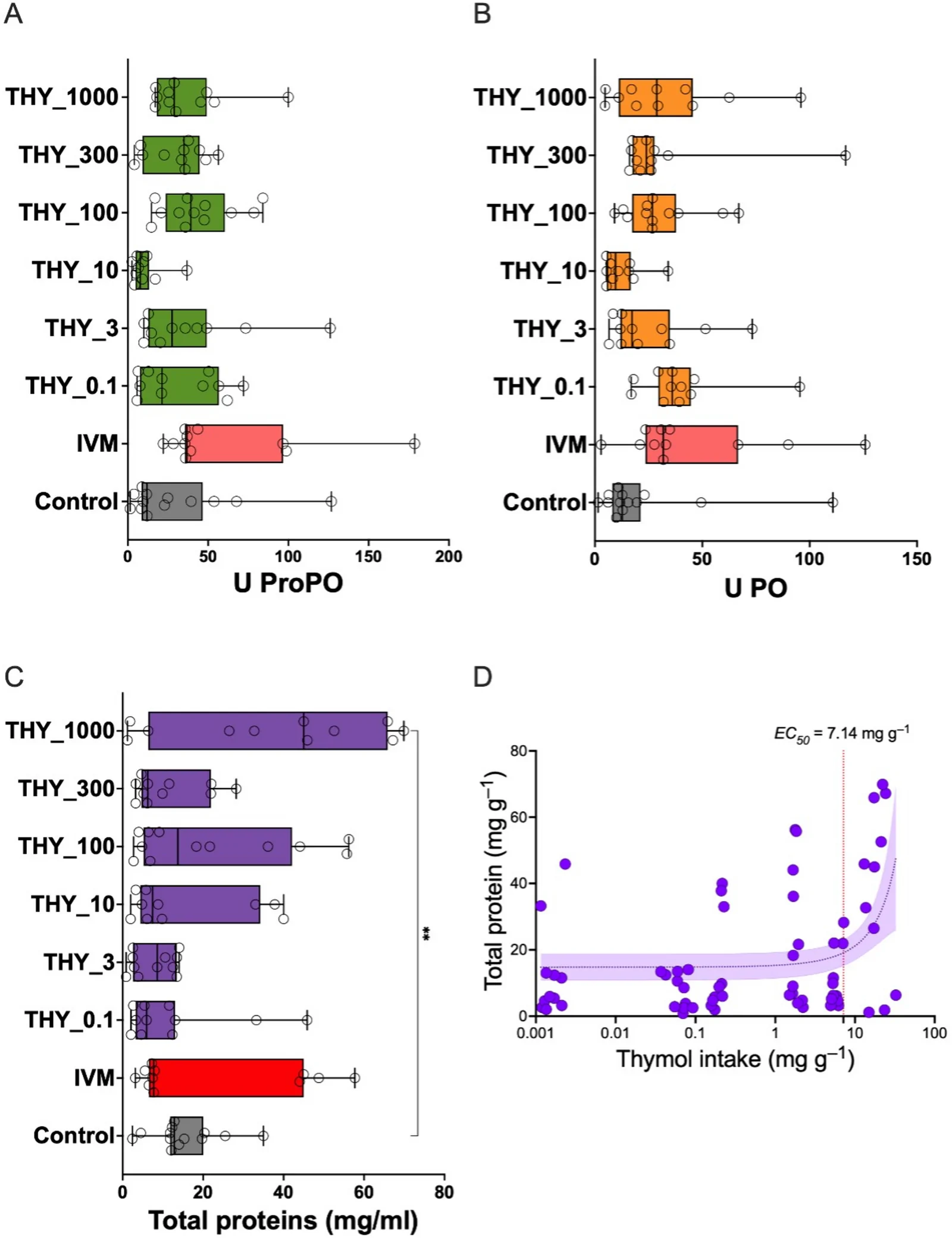
Effect of thymol intake on the immune system of *Thorectes lusitanicus*. **A** Prophenoloxidase (proPO) activity, (**B**) phenoloxidase (PO) activity, and (**C**) total protein content (mg ml⁻¹) in the haemolymph of individuals exposed to THY concentrations of 0.1, 3, 10, 100, 300, and 1000 mg kg⁻¹, compared to ivermectin (IVM; 100 µg kg⁻¹, positive control) and untreated dung (control; negative control). Colours represent the different compounds. Bars represent mean values ± standard deviation. Asterisks indicate significant differences compared to the control group (*P < 0.05; **P < 0.01; Dunnett’s post hoc test). **D** Concentration–response curve for the total protein content by THY intake. The shaded area represents the 95% confidence interval. The EC_50_ value indicates the amount of THY ingested per gram of individual required to increase protein levels by 50% relative to control values

In the case of proPO, although the ANOVA showed the existence of significant differences between some of the treatments (F_7,82_ = 2.27; *P* = 0.04), Dunnett’s post-hoc test showed that none of the THY concentrations significantly affected proPO levels. (Fig. 2A; Dunnett’s test: *P* > 0.05 for all comparisons). Ingestion of IVM at a sublethal concentration also did not significantly affect the proPO level (Fig. 2A; Dunnett’s test: *P* = 0.10). In the case of PO, the ANOVA showed no significant differences between any of the treatments (F_7,82_ = 1.67; *P* = 0.128), with no significant effect of THY intake at any concentration on PO levels compared to the control (Fig. 2B; Dunnett’s test: *P* > 0.05 for all comparisons). Ingestion of IVM also did not significantly affect the PO level (Fig. 2B; Dunnett’s test: *P* = 0.17). A significant increase in total protein concentration was detected only at the highest exposure level of THY (Figs. 2C and 1000 mg kg^–1^; *P* = 0.008). Correspondingly, the EC_50_ estimated from dose–response modelling was very high (7.14 g g⁻¹), exceeding the residue concentrations measured in dung under the conditions evaluated (Fig. 2D).

### Sublethal neurotoxic effects

In the pre-lethal ataxia assay, 45% of individuals exposed to ivermectin (IVM) exhibited clear signs of neurotoxicity, characterised by partial antennal paralysis and impaired locomotor coordination. In contrast, beetles exposed to THY did not display alterations in antennal reflexes or walking performance at any of the tested concentrations. Accordingly, the incidence of ataxia was 0% in all THY treatments, indicating an absence of observable neurotoxic effects under the experimental conditions.

## Discussion

In recent years, increasing evidence of sublethal and lethal effects of synthetic anthelmintics on non-target organisms, particularly dung beetles, has prompted the search for safer alternatives. In this context, the identification and evaluation of natural compounds with reduced ecological risk to dung beetles has become a priority in ecotoxicological research. The present study addresses this challenge by integrating residue analysis with physiological endpoints to assess the ecological safety of the phytochemical thymol (THY) in *Thorectes lusitanicus*, a functionally sensitive dung beetle species, thereby contributing to a growing body of evidence aimed at reconciling effective parasite control with biodiversity conservation in agroecosystems.

Field and laboratory studies have consistently shown that dung beetles are among the non-target organisms most vulnerable to residues of macrocyclic lactones (MLs) such as ivermectin (IVM), with tunnelling species frequently identified as the most vulnerable functional group when compared with roller or dweller species (Beynon et al. 2012; Jacobs and Scholtz 2015; Manning et al. 2018; Martínez et al. 2018; Sands and Wall 2018; Ishikawa and Iwasa 2020; Correa et al. 2022; Tovar et al. 2023). In agreement with previous reports identifying IVM as a potent neurotoxic agent, our results support its ecotoxic potential, as indicated by the 45% of individuals showing ataxic symptoms following exposure, consistent with previously reported impairments in locomotion and sensory function (Verdú et al. 2015, 2018b; González-Tokman et al. 2017). Such behavioural impairment is consistent with the neurotoxic mode of action described for ivermectin and has been associated with reduced fitness and altered ecosystem functioning in dung beetle assemblages (Verdú et al. 2015, 2018a; González-Tokman et al. 2017; Villada-Bedoya et al. 2019; Finch et al. 2020; Ambrožová et al. 2021; Mackenzie et al. 2022; Urrutia et al. 2025). Together, these findings reinforce the well-documented toxicity of IVM to dung beetles and highlight the substantial ecological risks associated with its widespread use in livestock systems.

In contrast, the present study indicates that THY is unlikely to induce ecotoxicologically relevant sublethal effects in *T. lusitanicus* under the exposure scenarios evaluated. Residue analysis indicated that following administration of the commercial feed additive NEXT ENHANCE^®^ 150, THY was excreted in faeces at consistently very low concentrations (0.260 mg kg^–1^), with no significant temporal variation. Consistent with previous trials conducted in different cattle systems, THY is excreted in bovine faeces at very low concentrations following administration of standard doses (Meeran et al. 2017; Kowalczyk et al. 2020; Biswal and Pazhamalai 2021; Hikal et al. 2021). These low excretion rates and trace residue levels are comparable to those reported in previous studies assessing phytochemical supplementation in livestock diets (Verdú et al. 2023), suggesting that such compounds exhibit limited environmental persistence and a low potential for accumulation in pasture ecosystems. In contrast, IVM is highly toxic at low concentrations, and it is frequently associated with high residue levels in cattle dung. These residues may persist and accumulate in soils and aquatic systems, thereby negatively affecting invertebrate communities and disrupting ecological dynamics (Canga et al. 2009; Mesa et al. 2017; Iglesias et al. 2018; Heinrich et al. 2021; de Souza and Guimarães 2022).

At the organismal level, ecotoxicological assays showed that thymol did not elicit measurable adverse effects on olfactory responses, immune system parameters or motor function in *T. lusitanicus* under the experimental conditions tested. In addition, no behavioural abnormalities were detected in THY-treated individuals at the concentrations tested. The estimated IC_50_ and EC_50_ values for both olfactory and immune endpoints indicate that relatively high intake thresholds would be required to induce measurable physiological alterations. For example, based on measured THY residues and the estimated IC_50_ threshold, the quantity of dung required to affect antennal response would greatly exceed typical individual consumption rates. However, the actual ecological relevance of these thresholds under field conditions remains uncertain and warrants further investigation, particularly studies assessing THY concentrations in faeces under different livestock health treatments and the feeding rates of diverse dung beetle species.

The results of this study complement and extend previous work conducted on other dung beetle taxa. Studies in *Ateuchetus cicatricosus*, a roller species, have similarly reported the absence of lethal or sublethal effects of THY and other phytochemicals, even at concentrations nearly 1,000-fold higher than realistic exposure levels (Verdú et al. 2023). The convergence of results across phylogenetically and functionally distinct dung beetles suggests that the low ecotoxicity of THY is not strongly species-specific, at least among the taxa evaluated to date. This is particularly relevant given that tunnelling dung beetles are frequently regarded as a conservative model for risk assessment due to their high exposure potential.

In conclusion, the widespread use of MLs anthelmintics continues to pose significant risks to non-target invertebrates and the ecosystem services they provide. In this study, we show that dietary exposure to THY does not induce ecotoxicologically relevant sublethal effects on olfactory function, immune parameters or motor performance in the tunneller dung beetle *T. lusitanicus*. Importantly, the physiological thresholds at which THY effects were detected were substantially higher than the residue levels measured in dung under the evaluated conditions. In contrast, IVM produced pronounced sensory impairment and neurotoxic symptoms at environmentally relevant concentrations, reinforcing its well-documented ecotoxic potential. Together, these findings indicate that THY is unlikely to pose significant ecotoxicological risks in *T. lusitanicus*. Although this species is considered a representative model of dung beetle, these results provide additional evidence supporting the safety of THY at therapeutic concentrations. While information on its effects across other taxa remains limited and further field-based studies are warranted, THY-based phytochemical anthelmintics may represent a promising strategy to reduce the environmental footprint of parasite control in livestock systems.

## Data Availability

The data that support the findings of this study are openly available in the University of Alicante Repository at http://hdl.handle.net/10045/161910.
